# GR-ConvNet v2: A Real-Time Multi-Grasp Detection Network for Robotic Grasping †

**DOI:** 10.3390/s22166208

**Published:** 2022-08-18

**Authors:** Sulabh Kumra, Shirin Joshi, Ferat Sahin

**Affiliations:** 1 The Department of Electrical Engineering, Rochester Institute of Technology, Rochester, NY 14623, USA; 2 eBots Inc., Fremont, CA 94539, USA; 3 Siemens Corporation, Berkeley, CA 94704, USA

**Keywords:** robotic manipulation, grasping, deep learning

## Abstract

We propose a dual-module robotic system to tackle the problem of generating and performing antipodal robotic grasps for unknown objects from the n-channel image of the scene. We present an improved version of the Generative Residual Convolutional Neural Network (GR-ConvNet v2) model that can generate robust antipodal grasps from n-channel image input at real-time speeds (20 ms). We evaluated the proposed model architecture on three standard datasets and achieved a new state-of-the-art accuracy of 98.8%, 95.1%, and 97.4% on Cornell, Jacquard and Graspnet grasping datasets, respectively. Empirical results show that our model significantly outperformed the prior work with a stricter IoU-based grasp detection metric. We conducted a suite of tests in simulation and the real world on a diverse set of previously unseen objects with adversarial geometry and household items. We demonstrate the adaptability of our approach by directly transferring the trained model to a 7 DoF robotic manipulator with a grasp success rate of 95.4% and 93.0% on novel household and adversarial objects, respectively. Furthermore, we validate the generalization capability of our pixel-wise grasp prediction model by validating it on complex Ravens-10 benchmark tasks, some of which require closed-loop visual feedback for multi-step sequencing.

## 1. Introduction

Robotic manipulators are constantly compared to humans due to the inherent characteristics of humans to instinctively grasp an unknown object rapidly and with ease based on their own experiences. As increasing research is being conducted to make robots more intelligent, there exists a demand for a generalized technique to infer fast and robust grasps for any kind of object that the robot encounters. The major challenge is being able to precisely transfer the knowledge that the robot learns to novel real-world objects.

In this work, we present a modular robot agnostic approach to tackle this problem of grasping unknown objects. We propose a Generative Residual Convolutional Neural Network (GR-ConvNet) that generates antipodal grasps for every pixel in an n-channel input image. We use the term generative to distinguish our method from other techniques that output a grasp probability or classify grasp candidates in order to predict the best grasp. We provide several experiments and ablation studies in both standard benchmarking datasets and real settings to evaluate the key components of the proposed system.

**Figure 1:**
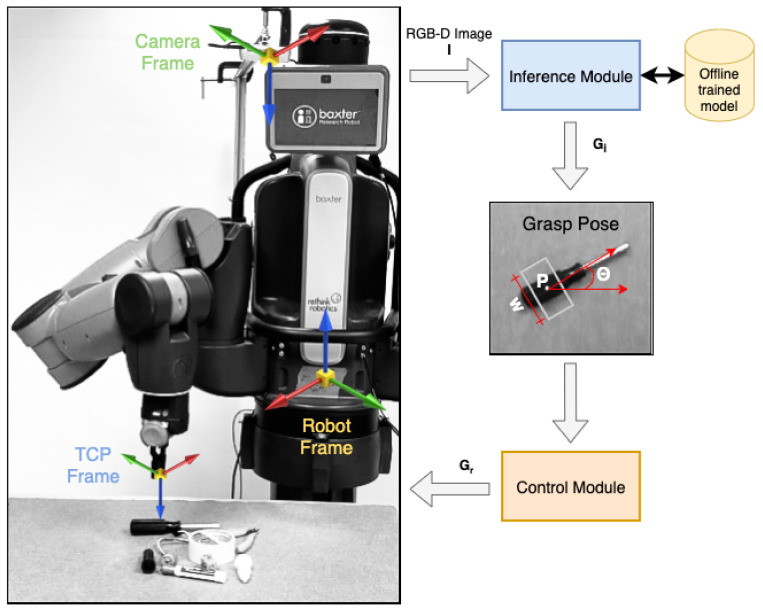
Overview. A real-time multi-grasp detection framework to predict, plan and perform robust antipodal grasps for the objects in the camera’s field of view using an offline trained GR-ConvNet model. Our system can grasp novel objects in isolation as well as in clutter. Video (accessed on 20 July 2022): https://youtu.be/cwlEhdoxY4U.

Figure 1 shows an overview of the proposed system architecture. It consists of two main modules: the inference module and the control module. The inference module acquires RGB and aligned depth images of the scene from the RGB-D camera. The images are pre-processed to match the input format of the proposed GR-ConvNet model trained on an offline grasping dataset. The network generates quality, angle, and width images, which are then used to infer antipodal grasp poses. The control module consists of a task controller that prepares and executes a plan to perform a pick and place task using the grasp pose generated by the inference module. It communicates the required actions to the robot through a ROS interface using a trajectory planner and controller.

In robotic grasping, it is very essential to generate grasps that are not just robust but also the ones that require the least amount of computation time. Our state-of-the-art technique demonstrates both of these from our outstanding results in generating robust grasps with the lowest recorded inference time of 20 ms on the Cornell Grasp dataset as well as the new Jacquard dataset. We also demonstrate that our technique works equally well in the real world with novel objects using a robotic manipulator. Unlike the previous work performed in robotic grasping [1,2,3,4], where the required grasp is predicted as a grasp rectangle calculated by choosing the best grasp from multiple grasp probabilities, our network generates three images from which we can infer grasp rectangles for multiple objects. Additionally, it is possible to infer multiple grasp rectangles for multiple objects from the output of GR-ConvNet in one shot thereby decreasing the overall computational time.

The key contributions of this work are:

- A dual-module robotic system that predicts, plans, and performs antipodal grasps for single or multiple objects in the scene. We open-sourced the implementation of the proposed inference and control modules.
- A novel Generative Residual Convolutional Neural Network (GR-ConvNet) architecture that predicts suitable antipodal grasp configurations for objects in the camera’s field of view at real-time speeds of 20 ms.
- We evaluate the generalization capabilities of the architecture and its prediction performance on publicly available grasping datasets and achieve a new state-of-the-art accuracy of 98.8%, 95.1%, and 97.4% on Cornell [5], Jacquard [6] and Graspnet [7] grasping datasets, respectively.
- An ablation study to understand the contribution of each component of the GR-ConvNet architecture and training process.
- Simulation experiments to evaluate the performance of GR-ConvNet trained on Cornell and Jacquard datasets with objects from Yale-CMU-Berkeley (YCB) object set [8] in isolated and cluttered scenarios. We demonstrated that GR-ConvNet performs significantly better than the GGCNN models [9] in both isolated and cluttered scenarios, with improvements of 12.5% and 14.5%, respectively.
- Real-world experiments with a 7 degree of freedom robotic manipulator to demonstrate that the proposed model can be deployed on a robotic manipulator to perform antipodal grasp at real-time speeds with a success rate of 95.4% and 93% on novel household and adversarial objects, respectively.
- Demonstration of the generalization capabilities of the proposed GR-ConvNet to various manipulation tasks by evaluating it on the Ravens-10 benchmark tasks presented by Zeng et al. in [10]. Furthermore, we validate that the sampling efficiency of GR-ConvNet is extremely impressive when evaluated on unseen test settings.

This journal paper is an extension of a conference paper that appeared in IEEE/RSJ International Conference on Intelligent Robots and Systems (IROS) 2020 [11]. This version introduces an improved version of the GR-ConvNet along with an ablation study in Section 6.5. In addition to Cornell and Jacquard datasets, this paper includes network performance evaluation on the Graspnet dataset in Section 6.4. It also includes additional simulation and real-world experiments and more system details in Section 7. In addition, we validate the flexibility and generalizability of the GR-ConvNet model to various vision-based manipulation tasks in Section 8. Other additional changes include details of the calibration procedure, visualizations and analysis of predicted grasps as compared to ground truth grasps, and illustrations of the clutter scene removal task in a real-world setup.

## 2. Related Work

**Figure 2:**
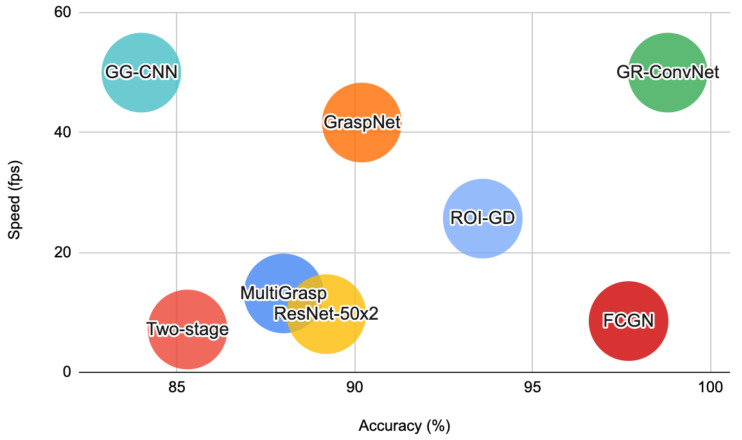
Performance comparison of GR-ConvNet on Cornell Grasping Dataset with prior work.

Our work lies at the intersection of robotic grasping, computer vision, and deep learning. In this section, we briefly review the related work in these domains. Table 1 provides a comparison of our work with recent related work in robotic grasping for unknown objects using learning-based approaches. Figure 2 shows the performance comparison of GR-ConvNet on Cornell Grasping Dataset with prior work in terms of speed and accuracy.

### 2.1. Robotic Grasping

There has been extensive ongoing research in the field of robotics, especially robotic grasping. Although the problem seems to just be able to find a suitable grasp for an object, the actual task involves multifaceted elements such as the object to be grasped, the shape of the object, physical properties of the object and the gripper with which it needs to be grasped among others. Early research in this field involved hand-engineering the features [17,18], which can be a tedious and time-consuming task but can be helpful for learning to grasp objects with multiple fingers such as [19,20].

Initially for obtaining a stable grasp, the mechanics and contact kinematics of the end effector in contact with the object were studied and the grasp analysis was performed as seen from the survey by [21,22]. Prior work [23] in robotic grasping for novel objects involved using supervised learning which was trained on synthetic data, but it was limited to environments such as offices, kitchens, and dishwashers. Satish et al. [24] introduced a Fully Convolutional Grasp Quality Convolutional Neural Network (FC-GQ-CNN) which predicted a robust grasp quality by using a data collection policy and synthetic training environment. This method enabled an increase in the number of grasps considered to 5000 times in 0.625 s. Bousmalis et al. [25] discussed domain adaptation and simulation in order to bridge the gap between simulated and real-world data. In that pixel-level domain adaptation model, GraspGAN was used to generate adapted images that are similar to real ones and are differentiated by the discriminator network. Trembley et al. [26] worked on a similar problem as Bousmalis et al., they used a deep network trained only on synthetic images with 6 DoF pose of known objects. However, this has been shown to work with household items only. James et al. [27] discuss a Randomized to Canonical Adaptation Networks (RCANs) method that learns to translate images from randomized simulated environments to their equivalent simulated canonical images using an image-conditioned GAN. They then use this to train their RL algorithm for real-world images. Furthermore, an actor–critic network that combines the results obtained by the actor network is presented in [28] which samples grasp samples directly with the results obtained from a critic network that re-scores the results obtained from the actor network to find stable and robust grasps. However, the current research relies more on using the RGB-D data to predict grasp poses. These approaches depend wholly on deep learning techniques.

### 2.2. Deep Learning for Grasping

Deep learning has been a hot topic of research since the advent of ImageNet success and the use of GPUs and other fast computational techniques. Moreover, the availability of affordable RGB-D sensors enabled the use of deep learning techniques to learn the features of objects directly from image data. Recent experimentations using deep neural networks [2,29,30] have demonstrated that they can be used to efficiently compute stable grasps. Pinto et al. [3] used an architecture similar to AlexNet which shows that by increasing the size of the data, their CNN was able to generalize better to new data. Varley et al. [31] propose an interesting approach to grasp planning through shape completion where a 3D CNN was used to train the network on the 3D prototype of objects on their own dataset captured from various viewpoints. Guo et al. [32] used tactile data along with visual data to train a hybrid deep architecture. Mahler et al. [33] proposed a Grasp Quality Convolutional Neural Network (GQ-CNN) that predicts grasps from synthetic point cloud data trained with Dex-Net 2.0 grasp planner dataset. Levine et al. [34] discuss the use of monocular images for hand-to-eye coordination for robotic grasping using a deep learning framework. They use a CNN for grasp success prediction and further use continuous servoing to continuously servo the manipulator to correct mistakes. Antanas et al. [35] discuss an interesting approach known as a probabilistic logic framework that is said to improve the grasping capability of a robot with the help of semantic object parts. This framework combines high-level reasoning with low-level grasping. The high-level reasoning comprises object affordances, its categories, and task-based information while low-level reasoning uses visual shape features. This has been observed to work well in kitchen-related scenarios.

### 2.3. Grasping Using Uni-Modal Data

Johns et al. [36] used a simulated depth image to predict a grasp outcome for every grasp pose predicted and select the best grasp by smoothing the predicted pose using a grasp uncertainty function. A generative approach to grasping is discussed by Morrison et al. [9]. The Generative grasp CNN architecture generates grasp poses using a depth image and the network computes grasp on a pixel-wise basis. Morrison et al. [9] suggests that it reduces existing shortcomings of discrete sampling and computational complexity. Another recent approach that merely relies on depth data as the sole input to the deep CNN is as seen in [29].

### 2.4. Grasping Using Multi-Modal Data

There are different ways of handling objects in multi-modalities. Many have used separate features to learn the modalities which can be computationally exhaustive. Wang et al. [12] proposed methods that consider multi-modal information as the same. Jiang et al. [5] used RGB-D images to infer grasps based on a two-step learning process. The first step was used to narrow down the search space and the second step was used to compute the optimal grasp rectangle from the top grasps obtained using the first method. Lenz et al. [1] used a similar two-step approach but with a deep learning architecture, which, however, could not work well on all types of objects and often predicted a grasp location that was not the best grasp for that particular object such as in [5] the algorithm predicted grasp for a shoe was from its laces which in practice failed when the robot tried to grasp using the shoelaces while in [1] the algorithm sometimes could not predict grasps which are more practical using just the local information as well as due to the RGB-D sensor used. Yan et al. [37] used a point cloud prediction network to generate a grasp by first preprocessing the data by obtaining the color, depth, and masked images and then obtaining a 3D point cloud of the object to be fed into a critic network to predict a grasp. Chu et al. [13] propose a novel architecture that can predict multiple grasps for multiple objects simultaneously rather than for a single object. For this, they used a multi-object dataset of their own. The model was also tested on the Cornell Grasp Dataset. A robotic grasping method that consists of a ConvNet for object recognition and a grasping method for manipulating the objects is discussed by Ogas et al. [38]. The grasping method assumes an industry assembly line where the object parameters are assumed to be known in advance. Kumra et al. [4] proposed a Deep CNN architecture that uses residual layers for predicting robust grasps. The paper demonstrates that a deeper network along with residual layers learns better features and performs faster. Asif et al. [39] introduced a consolidated framework known as EnsembleNet in which the grasp generation network generates four grasp representations and EnsembleNet synthesizes these generated grasps to produce grasp scores from which the grasp with the highest score gets selected.

### 2.5. 6-DoF Grasping

The 3-DOF grasp representation constrains the gripper pose to be parallel to the RGB image plane, which can be a challenge when grasping objects from a dense clutter. To overcome this, Liang et al. proposed PointNetGPD, which can directly process the 3D point cloud that locates within the gripper for grasp evaluation [40]. Similarly, Mousavian et al. introduced a 6-DOF GraspNet, which is a grasp evaluator network that maps a point cloud of the observed object and the robot gripper to a quality assessment of the 6D gripper pose. Moreover, they demonstrated that the gradient of GraspNet can be used to move the gripper out of collision and ensure that the gripper is well aligned with the object [41]. Murali et al. proposed a method that plans 6-DOF grasps for objects in a cluttered scene from partial point cloud observations. Their learned collision checking module was able to provide effective grasp sequences to retrieve objects that were not immediately accessible [42]. The two step deep geometry-aware grasping network (DGGN) proposed by Yan et al. first learns to build the mental geometry-aware representation by reconstructing the scene from RGB-D input, and then learns to predict grasp outcome with its internal geometry-aware representation. The outcome of the model is used to sequentially propose grasping solutions via analysis-by-synthesis optimization [43]. A large-scale benchmark for object grasping called GraspNet-1Billion along with an end-to-end grasp pose prediction network to learn the approaching direction and operation parameters in a decoupled manner is introduced in [7].

## 3. Problem Formulation

In this work, we define the problem of robotic grasping as predicting antipodal grasps for unknown objects from an n-channel image of the scene and executing it on a robot.

Instead of the five-dimensional grasp representation used in [1,2,4], we use an improved version of the grasp representation similar to the one proposed by Morrison et al. in [9]. We denote the grasp pose in the robot frame as:

(1) $$G_{r}=\left(P,\Theta_{r},W_{r},Q\right)$$

where, P=(x,y,z) is tool tip’s center position, $\Theta_{r}$ is tools rotation around the z-axis, $W_{r}$ is the required width for the tool, and *Q* is the grasp quality score.

We detect a grasp from an n-channel image $I\in R^{n\times h\times w}$ with height *h* and width *w*, which can be defined as:

(2) $$G_{i}=\left(u,v,d,\Theta_{i},W_{i},Q\right)$$

where (u,v) corresponds to the center of grasp in image coordinates, *d* is the depth value, $\Theta_{i}$ is the rotation in the camera’s frame of reference, $W_{i}$ is the required width in image coordinates, and *Q* is the same scalar as in Equation (1).

The grasp quality score *Q* is the quality of the grasp at every point in the image and is indicated as a score value between 0 and 1, where a value that is in proximity to 1 indicates a greater chance of grasp success. $\Theta_{i}$ indicates the antipodal measurement of the amount of angular rotation required at each point to grasp the object of interest and is represented as a value in the range $\left[\frac{-\pi }{2},\frac{\pi }{2}\right]$. $W_{i}$ is the required width which is represented as a measure of uniform depth and indicated as a value in the range of $\left[0,W_{max}\right]$ pixels. $W_{max}$ is the maximum width of the antipodal gripper.

To execute a grasp obtained in the image space on a robot, we can apply the following transformations to convert the image coordinates to the robot’s frame of reference.

(3) $$G_{r}=T_{rc}\left(T_{ci}\left(G_{i}\right)\right)$$

where, $T_{ci}$ is a transformation that converts image space into the camera’s 3D space using the intrinsic parameters of the camera, and $T_{rc}$ converts camera space into the robot space using the camera pose calibration value.

This notation can be scaled for multiple grasps in an image. The collective group of all the grasps can be denoted as:

(4) $$G=\left(\Theta ,W,Q\right)\in R^{3\times h\times w}$$

where Θ,W, and Q represents three images in the form of grasp angle, grasp width, and grasp quality score, respectively, calculated at every pixel of an image using Equation (2).

## 4. Proposed Approach

In this section, we describe our proposed dual-module system to predict, plan and perform antipodal grasps for novel objects in the scene. The overview of the proposed system is shown in Figure 1. The inference module is used to predict grasp poses in the image frame ($G_{i}$) for the objects in the camera’s field of view. The control module converts these grasp poses into robot frames ($G_{r}$) and then plans and executes robot trajectories to perform antipodal grasps.

### 4.1. Inference Module

**Figure 3:**
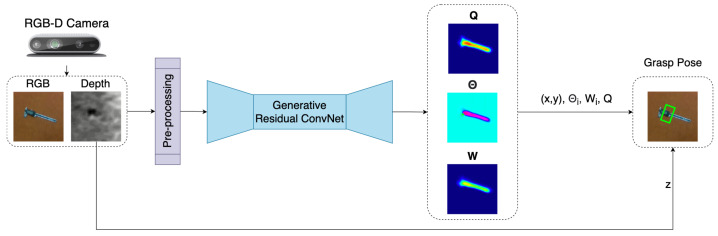
Inference module predicts suitable grasp poses for the objects in the camera’s field of view.

Figure 3 shows the inference module, which consists of three parts: image pre-processing, generation of pixel-wise grasp using GR-ConvNet v2, and computation of grasp pose(s). The input data is first pre-processed where it is cropped, resized, and normalized to suit the input requirements of GR-ConvNet. If the input has a depth image, it is inpainted to obtain a depth representation [44]. The 224×224 n-channel processed input image is fed into the GR-ConvNet v2. It uses n-channel input that is not limited to a particular type of input modality such as a depth-only or RGB-only image as our input image. Thus, making it generalized for any kind of input modality. The GR-ConvNet generates pixel-wise grasp in the form of grasp angle Θ, grasp width **W**, and grasp quality score **Q** as the output using the features extracted from the pre-processed image.

The three output images are utilized to infer grasp poses in the image frame ($G_{i}$) using Equation (2). In the case of a single grasp prediction, the pixel with maximum value in **Q** is identified and the corresponding pixel location is used as (u,v) and pixel value is used a *Q*. The same pixel locations in Θ, **W** and depth frame are used to determine $\Theta_{i}$, $W_{i}$, and *d*, respectively. For multi-grasp prediction, local peaks are determined in **Q** using [45] to calculate all grasp poses.

### 4.2. Control Module

**Figure 4:**
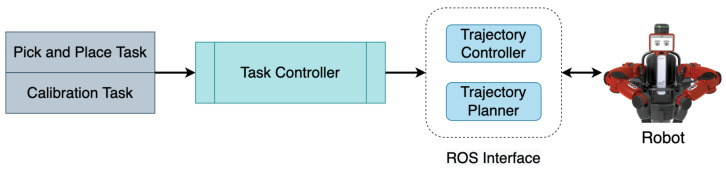
Control module uses the grasp poses generated by the inference module to plan and execute robot trajectories to perform antipodal grasps.

The control module mainly incorporates a task controller that performs tasks such as pick-and-place and calibration. The architecture of the control module is shown in Figure 4. The task controller requests a grasp pose from the inference module, which returns the grasp pose with the highest quality score. The grasp pose is then converted from the camera frame into the robot frame using Equation (3) and the transform is calculated from an automatic hand-eye calibration process described in Section 7.3. Further, the grasp pose in the robot frame ($G_{r}$) is used to plan a collision-free trajectory to perform the pick and place action using inverse kinematics through a ROS interface. The robot then executes the planned trajectory. Due to our modular approach and automatic hand-eye calibration process, this system can be adapted for any robotic manipulator and camera setup.

## 5. Generative Residual Convolutional Neural Network

Deep learning has redefined how robotic grasping was approached in the past. Further, CNNs have enhanced the way object detection and classification problems have been dealt with in computer vision. Furthermore, state-of-the-art results have been obtained by using residual networks for deeper architectures [4,14]. These two deep learning techniques are the building blocks of our novel architecture. In this section, we present an improved Generative Residual Convolutional Neural Network (GR-ConvNet v2) to approximate the complex function $I\overset{f_{\theta }}{\to }G_{i}$, where $f_{\theta }$ denotes a neural network with θ being the weights.

### 5.1. Network Architecture

**Figure 5:**
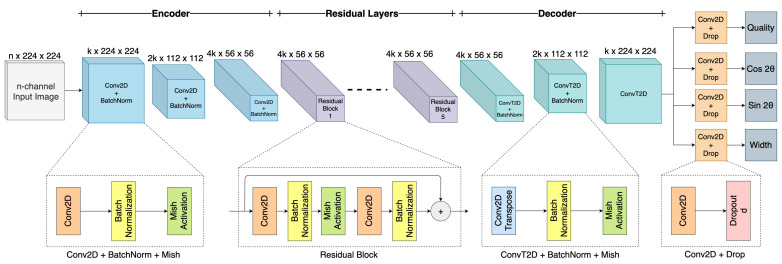
Network architecture of the Generative Residual Convolutional Neural Network v2, where n is the number of input channels, k is the number of filters, and d is the dropout rate. The network takes in an n-channel input image of size 224×224 and generates pixel-wise grasps in the form of grasp quality, grasp angle and grasp width.

Figure 5 shows the proposed GR-ConvNet v2 model, which is a generative architecture that takes in an n-channel input image of size 224×224 and generates pixel-wise grasps in the form of four images of the same size. These output images consist of grasp quality score **Q**, required angle Θ in the form of cos2Θ, and sin2Θ, as well as the required width **W** of the end effector. Since the antipodal grasp is uniform around $\pm \frac{\pi }{2}$, we extract the angle in the form of two elements cos2Θ and sin2Θ that output distinct values that are combined to form the required angle.

The network consists of three parts: encoder, residual layers, and decoder. The n-channel image is passed through the encoder which consists of three convolutional layers, followed by five residual layers, and the decoder which consists of three convolution transpose layers to generate four images. The convolutional layers with a filter size of k extract the features from the input image. The output of the convolutional layer is then fed into five residual layers. As we know, accuracy increases with increasing the number of layers. However, it is not true when you exceed a certain number of layers, which results in the problem of vanishing gradients and dimensionality error, thereby causing saturation and degradation in the accuracy. Thus, using residual layers enables us to better learn the identity functions by using skip connections [46]. After passing the image through these convolutional and residual layers, the size of the image is reduced to 56×56, which can be difficult to interpret. Therefore, to make it easier to interpret and retain spatial features of the image after convolution operation, we up-sample the image by using a convolution transpose operation. Thus, we obtain the same size of the image at the output as the size of the input. In this improved version of the network, as compared to [11], we added a dropout layer after each of the outputs for regularization that favors rare but useful features. We also replaced the ReLU activation function with Mish throughout the network, which delivered across-the-board improvements in training stability. We believe that the slight allowance for negative values in the Mish activation function allows for better gradient flow compared to the hard zero bound in ReLU.

Our network has only 1.9 million parameters with k = 32 and n = 4, which indicates that our network is comparatively shorter as opposed to other networks [4,14,39]. Thereby making it computationally less expensive and faster in contrast to other architectures using similar grasp prediction techniques that contain millions of parameters and complex architectures. The lightweight nature of our model makes it suitable for closed-loop control at a rate of up to 50 Hz.

### 5.2. Training Methodology

For a dataset having objects $D=\left\{D_{1}\cdots D_{n}\right\}$, input scene images $I=\left\{I^{1}\cdots I^{n}\right\}$ and ground truth grasp labels in image frame $\hat{G_{i}}=\left\{g_{1}^{1}\cdots g_{m_{1}}^{1}\cdots g_{1}^{2}\cdots g_{m_{n}}^{n}\right\}$, we train the proposed GR-ConvNet model end-to-end to learn the mapping function $f:\left(I,D\right)\to G_{i}$, where $G_{i}$ is the grasp generated by the network in image frame.

We analyzed the performance of various loss functions for our network and after running a few trials found that in order to handle exploding gradients, the smooth L1 loss also known as Huber loss works best. We define our loss as:

(5) $$L\left(y_{i},\hat{y_{i}}\right)=\frac{1}{n}\sum_{i}^{N}\mathrm{SmoothL}1\left(y_{i}-\hat{y_{i}}\right)$$

where SmoothL1 is given by:

(6) $$\mathrm{SmoothL}1\left(x\right)=\left\{\begin{array}{cc} 0.5x^{2}, & if\left|x\right|<1\\ \left|x\right|-0.5 & otherwise \end{array}\right.$$

and $y_{i}\in \left(Q,\Theta_{cos}\Theta_{sin},W\right)$ is the image generated by the model and $\hat{y_{i}}$ is the ground truth image. The overall loss function denoted in Equation (7) is a combined loss of the four output images generated by the model, which are in the form of quality, angle in cos and sin, and required width.

(7) $$L=L_{quality}+L_{cos}+L_{sin}+L_{width}$$

We improved the training pipeline, as compared to [11], by training the models using Ranger optimizer [47] instead of the Adam optimizer [48]. Ranger combines two latest breakthroughs in deep learning optimizers that builds on top of Adam—Rectified Adam, and LookAhead. Training with Rectified Adam gets off to a solid start intrinsically by adding in a rectifier that dynamically tamps down the adaptive learning rate until the variance stabilizes [49]. LookAhead lessens the need for extensive hyperparameter tuning while achieving faster convergence across different deep learning tasks with minimal computational overhead [50].

**Figure 6:**
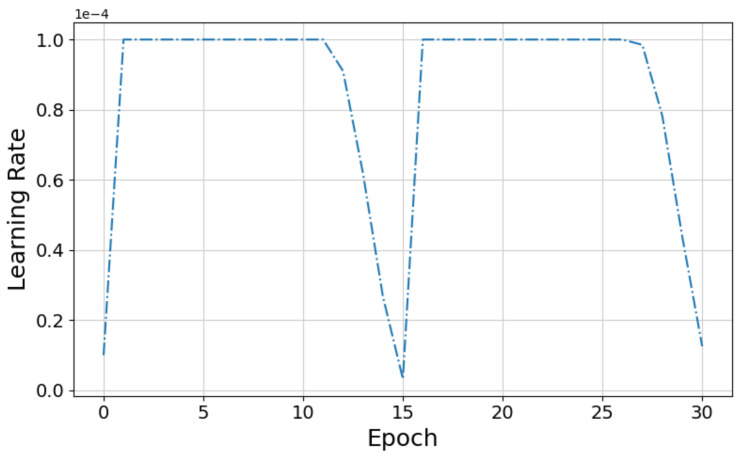
Flat + Cosine anneal learning rate curve used for training.

Instead of keeping the learning rate fixed at $10^{-3}$ throughout the training process, as in [11], we used the Flat + Cosine anneal as ramp-up and ramp-down curve for the learning rates during training. The learning rate is kept constant at $10^{-4}$ for first few epochs and then annealed to the target learning rate of $10^{-7}$ according to the law of cosine learning rate [51]. The ramp up and ramp-down cycle is down twice during training as illustrated in Figure 6.

### 5.3. Grasp Detection Metric

For a fair comparison of our results, we use the rectangle metric [5] proposed by Jiang et al. to report the performance of our system. According to the proposed rectangle metric, a grasp is considered valid when it satisfies the following two conditions:

- The Jaccard index or intersection over union (IoU) score between the ground truth grasp rectangle and the predicted grasp rectangle is more than 25%.
- The offset between the grasp orientation of the predicted grasp rectangle and the ground truth rectangle is less than $30^{\circ }$.

This IoU-based metric requires a grasp rectangle representation, but our model predicts image-based grasp representation $\hat{G_{i}}$ using Equation (2). Therefore, in order to convert from the image-based grasp representation to the rectangle representation, the value corresponding to each pixel in the output image is mapped to its equivalent rectangle representation similar to [9].

## 6. Network Evaluation

We evaluate GR-ConvNet v2 on three publicly available datasets to examine the outcome for each of the datasets based on factors, such as the size of the dataset, type of training data, and demonstrate our model’s capacity to generalize to any kind of object. We trained the model using three random seeds and reported the average of the three seeds. The execution times for our proposed model are measured on a system running Ubuntu 18.04 with an Intel Core i7-7800X CPU clocked at 3.50 GHz and an NVIDIA GeForce GTX 1080 Ti graphics card with CUDA 11.

**Figure 7:**
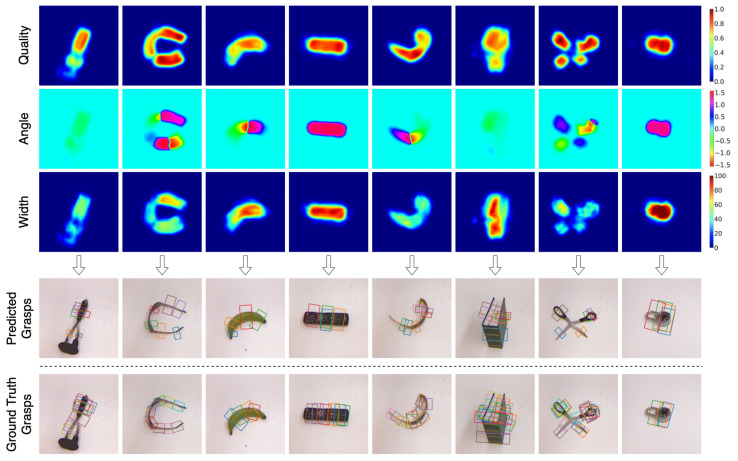
Qualitative results on Cornell Grasping Dataset. The top three rows (quality, angle and width) are the output of GR-Convnet. The bottom two rows are the predicted and ground truth grasps in rectangle grasp representation.

**Figure 8:**
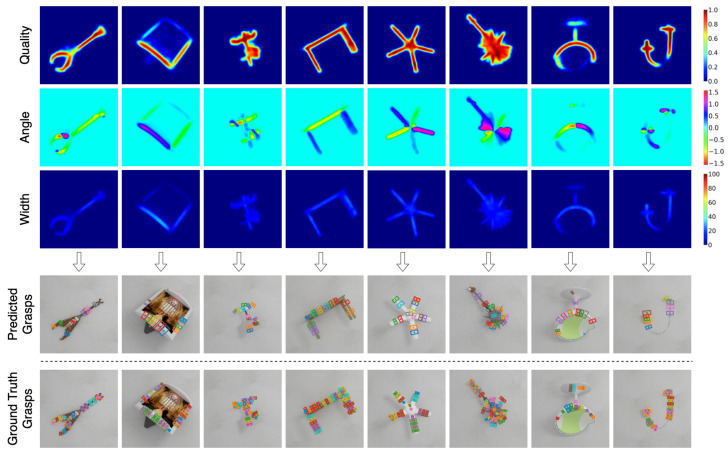
Qualitative results on Jacquard Grasping Dataset. The top three rows (quality, angle and width) are the output of GR-Convnet. The bottom two rows are the predicted and ground truth grasps in rectangle grasp representation.

**Figure 9:**
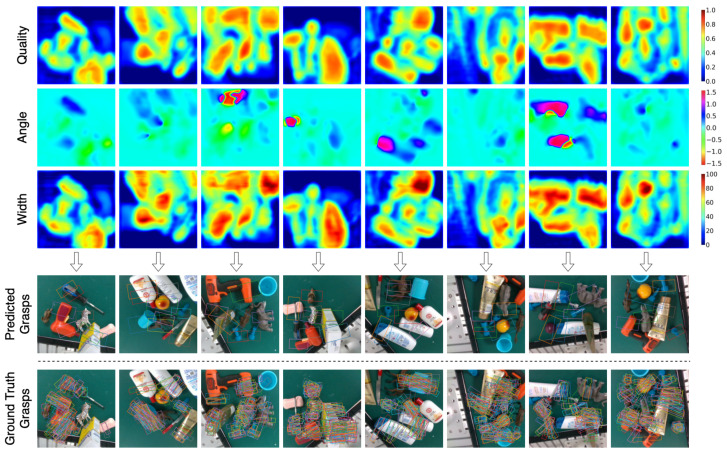
Qualitative results on Graspnet 1-billion Dataset. The top three rows (quality, angle and width) are the output of GR-Convnet. The bottom two rows are the predicted and ground truth grasps in rectangle grasp representation.

Figure 7, Figure 8 and Figure 9 shows the qualitative results obtained on previously unseen objects in Cornell, Jacquard, and Graspnet grasping datasets, respectively. The figure consists of output in the image representation $G_{i}$ in the form of grasp quality score *Q*, the required angle for grasping $\Theta_{i}$, and the required gripper width $W_{i}$. It also includes the output in the form of a rectangle grasp representation projected on the RGB image and the ground truth grasps.

### 6.1. Datasets

There are a limited number of publicly available antipodal grasping datasets. Table 2 shows a summary of the publicly available antipodal grasping datasets. We used three of these datasets for training and evaluating our model. The first one is the Cornell grasp dataset [5], which is the most common grasping dataset used to benchmark results, the second is a simulation Jacquard grasping dataset [6], which is more than 50 times bigger than the Cornell grasp dataset, and the third one is the more recent Graspnet 1-billion dataset [7].

#### 6.1.1. Cornell Grasp Dataset

The extended version of Cornell Grasp Dataset comprises 1035 RGB-D images with a resolution of 640×480 pixels of 240 different real objects with 5110 positive and 2909 negative grasps. The annotated ground truth consists of several grasp rectangles representing grasping possibilities per object. However, it is a small dataset for training our GR-ConvNet model; therefore, we create an augmented dataset using random crops, zooms, and rotations which effectively has 51k grasp examples. Only positively labeled grasps from the dataset were considered during training.

#### 6.1.2. Jacquard Grasping Dataset

The Jacquard Grasping Dataset is built on a subset of ShapeNet which is a large CAD model dataset. It consists of 54k RGB-D images with a resolution of 1024×1024 pixels and annotations of successful grasping positions based on grasp attempts performed in a simulated environment. In total, it has 1,181,330 unique grasp annotations for 11,619 distinct objects in simulation.

#### 6.1.3. Graspnet 1-Billion Dataset

Graspnet is a large-scale benchmark dataset that contains 190 cluttered and complex scenes captured by Kinect Azure and RealSense D435 cameras. In total, it contains 97,280 RGB-D images with over 1.1 billion grasp poses of 88 different objects. To use the raw rectangular images with a resolution of 1280×720 pixels, a square image of size 720×720 pixels is cropped around the mean center of the ground truth bounding box. Graspnet consists of 190 scenes, and each includes 256 images with annotations.

### 6.2. Evaluation on Cornell Dataset

We follow a cross-validation setup as in previous works [1,2,4,15,32], using image-wise, and object-wise data splits. The image-wise data split means that the training and validation sets are divided randomly, whereas the object-wise data split means that the objects in the validation set do not appear in the training set. Table 3 shows the performance of our method in comparison with other techniques used for grasp prediction. We obtained state-of-the-art accuracy of 98.8% on image-wise split and 97.7% on object-wise split using the improved GR-ConvNet model, outperforming all competitive methods as seen in Table 3. The results obtained on the previously unseen objects in the dataset depict that our network can predict robust grasps for different types of objects in the validation set. The data augmentation performed on the Cornell grasp dataset improved the overall performance of the network. Furthermore, the recorded prediction speed of 20 ms per image suggests that GR-ConvNet is suitable for real-time closed-loop applications.

We also evaluate the accuracy of our trained model at higher IoU thresholds. Table 4 contains the comparison of outcomes for the Cornell Dataset at different Jaccard thresholds. In contrast to the prior work [13,32,58], our approach maintains a high prediction accuracy even if the grasp detection metric is stricter. Our model outperforms the network proposed in [13] by 14% and in [58] by 11% at 40% IoU threshold.

### 6.3. Evaluation on Jacquard Dataset

For the Jacquard dataset, we trained our network on 90% of the dataset images and validated it on 10% of the remaining dataset. As the Jacquard dataset is much larger than the Cornell dataset, no data augmentation was required. Table 5 compares the results of our network with other methods on the Jacquard dataset. We used the IoU metric for grasp evaluation and observed an accuracy of 95.1% using GR-ConvNet v2 with RGB-D data as the input and a batch size of 16.

Depierre et al. released a web-based Simulated Grasp Trails (SGT) system to upload the scene index and corresponding grasp prediction [6]. The system rebuilds the scene in simulation and the grasp is executed by the simulated robot. The results of the execution are emailed to the user. We report these results in Table 5. Our results are the new state-of-the-art with an accuracy of 91.4% using the SGT metric.

### 6.4. Evaluation on Graspnet Dataset

The Graspnet dataset is gigantic, and the load on the computer when loading the immense amounts of grasps can cause problems. We reduced the load on computing resources by reducing the number of ground truth labels loaded per scene and pre-processing the dataset. Each grasp label in the Graspnet dataset has a quality measure associated with it, which is measured based on the friction coefficient μ. We discarded the ground-truth labels that are outside the cropped image and have poor grasp quality (μ<0.4).

In addition to the 5-fold cross-validation split (similar to the one used for the Cornell dataset), we use the predesignated data provided with the Graspnet dataset for training and testing. There are a total of 190 scenes. The first 100 scenes are used for training, and the testing data has been split into three categories: (i) objects already seen (scenes 101–130), (ii) objects similar to training (scenes 131–160), and (iii) objects not seen before (scenes 161–190). The validation results compared to prior work are summarized in Table 6 for the three testing splits provided with the dataset and the 5-fold cross-validation method. It can be seen that the proposed GR-ConvNet outperforms the state-of-the-art counterparts [9,13] by a large margin, with an improvement of 14.2% for the novel test set, which demonstrates its effectiveness in handling unseen scenarios.

### 6.5. Ablation Study

To better understand the performance of our model, a series of experiments were performed by tweaking a number of parameters including filter size, batch size, learning rate, and varying the number of layers. After evaluating the performance of multiple parameters, we determined the architecture that gave us the highest grasp prediction accuracy along with the lowest recorded inference time. This section discusses these experiments and elaborates on the contributions of each of the individual components and parameters that were chosen during our network design by evaluating the model on the Cornell dataset.

**Figure 10:**
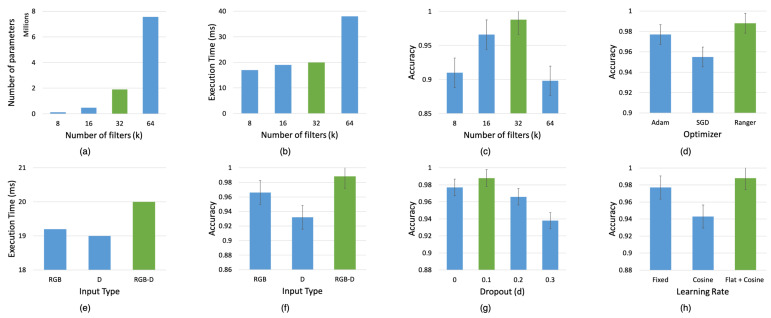
Ablation study results for GR-Convnet by training on different filter sizes (k), input channels (n), dropout (d), optimizers and learning rates. The model is evaluated using the 25% IoU metric against the Cornell dataset. Green indicates the selected parameter. (**a**–**c**) Number of parameters, execution time and accuracy for different number of filters. (**d**) Grasp prediction accuracy for different optimizers. (**e**–**f**) Execution time and prediction accuracy of the network for different modalities. (**g**) Grasp prediction accuracy for different dropout percentage. (**h**) Grasp prediction accuracy for different learning rates.

Firstly, we evaluated our network by varying the number of filters (k) at each layer as shown in Figure 10a–c. It can be seen from the figure that varying the number of filters plays a significant role in determining the accuracy of the network. We found that by increasing the number of filters (k), the accuracy increased proportionately until it reached a certain value and then started decreasing substantially. At this point, we also observed that the number of parameters and execution time increased drastically. In comparison, increasing the number of filters had little impact on the execution time as opposed to providing higher accuracy. However, the accuracy dropped when the number of filters (k) was increased beyond k = 32 while also increasing the number of parameters and execution time. Thus, we chose the set of parameters indicated in green, that yielded the maximum accuracy in comparison to an increased number of parameters and execution time.

Furthermore, we evaluated the performance of our network on different input modalities. The modalities that the model was tested on included uni-modal input such as depth only and RGB only input images; and multi-modal input such as RGB-D images. Figure 10e–f shows the performance of the network on different modalities. While RGB-only input data had lower execution times than the RGB-D input data, it had lower accuracy than the RGB-D input. The depth-only input data had the lowest execution times and the lowest accuracy compared to the RGB and the RGB-D input data. Thus, we observed that our network performed better on multi-modal data in comparison to uni-modal data since multiple input modalities enabled better learning of the input features.

Additionally, to study the effect of regularization on our network, we added dropout layers after the deconvolution layers. We tested the model with a dropout of 10%, 20%, and 30% feature drop against no dropout. From Figure 10g we can see that a dropout of 10% bumped the accuracy from 97.7% to 98.8% and a dropout of 20% and 30% reduced the accuracy below 97.7%. From the results, we can see that the model was slightly overfitting, and by dropping 10% of the features during training, we achieved an increase in the success rate by 1% on the validation set.

Finally, we studied the impact of different optimizers and learning rates discussed in Section 5.2 on the grasp prediction accuracy. Figure 10d shows that the Ranger optimizer improved the accuracy by 3.3% compared to the standard SGD optimizer. We also observed an improvement of 1.1% accuracy (shown in Figure 10h) when the model is trained using Flat + Cosine anneal as a ramp-up and ramp-down curve for the learning rates instead of a fixed learning rate as in [9,11].

## 7. Antipodal Grasping Using GR-ConvNet

In this section, we discuss our antipodal robotic grasping experiments and the results. Along with the state-of-the-art results on two standard datasets, we also demonstrate that our system equally outperforms in robotic grasping experiments for novel real-world objects. Furthermore, we show that our model can not only generate a single grasp for isolated objects but also multiple grasps for multiple objects in clutter. For a fair comparison, we implement an open-loop grasping method similar to previous work ([1,3,33]) and evaluate our approach on: (i) household objects, (ii) adversarial objects and (iii) objects in clutter.

### 7.1. Simulation Setup

**Figure 11:**
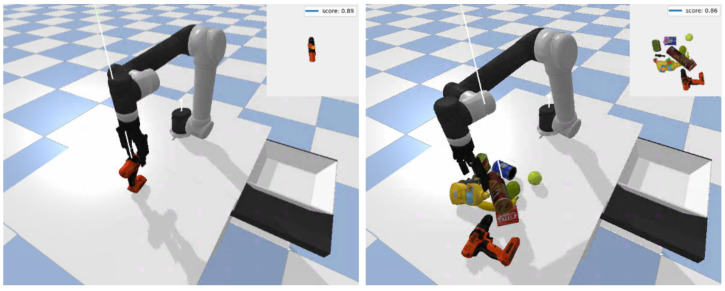
Examples of antipodal grasping in simulation. (**Left**) Grasping YCB objects in isolation. (**Right**) Grasping YCB objects in clutter.

To evaluate antipodal robotic grasping in simulation, we developed a simulation environment (show in Figure 11) in PyBullet [59], where a UR5e with a Robotiq 2F-140 antipodal gripper perceived the environment using an RGB-D camera looking over the robot’s workspace. Simulated objects from the Yale-CMU-Berkeley (YCB) object set [8], a benchmarking object set for robotic grasping, are used for the simulation experiments. At the beginning of each experiment, the robot is set to a predefined pose, and randomly selected object(s) are placed in an arbitrary pose(s) inside the robot’s workspace. In all experiments, the robot knows in advance about the placement pose in a basket, while the GR-ConvNet model needs to predict the best graspable pose for the given scene and send it to the robot to grasp the object, pick it up, and put it in the placement basket. A particular grasp is recorded as a success if the object is inside the basket at the end of the pick and place mission.

### 7.2. Simulation Experiments

We evaluated the performance of GR-ConvNet trained on Cornell and Jacquard in two different scenarios: isolated and cluttered. For the isolated object scenario, a randomly selected object is placed in an arbitrary pose inside the robot’s workspace and the robot executed the pick and place mission. In the case of the cluttered scenario, to generate a simulated scene containing a cluttered pile of objects, 10 objects are randomly spawned into a box placed on top of the table. The box is removed once all objects become stable, and then the robot repeatedly executes pick and place missions until there are no objects left in the robot’s workspace.

To report the performance of the model, we measure the pick success rate, which is the ratio of the number of successful grasps and the number of attempts. For each experiment, we ran a total of 200 grasp attempts and reported the pick success rate. Table 7 summarizes the results for different models tested with objects in isolation and clutter. It can be seen that the proposed GR-ConvNet performs significantly better than the GGCNN models in both isolated and cluttered scenarios, with improvements of 12.5% and 14.5%, respectively.

### 7.3. Real-World Setup

The real-world experiments were conducted on the 7-DoF Baxter Robot by Rethink Robotics. A two-fingered parallel gripper was used for grasping the test objects. The Intel RealSense Depth Camera D435, which uses stereo vision to calculate depth, was used to obtain the scene image. The image bundle consists of a pair of RGB sensors, depth sensors, and an infrared projector. The camera was statically mounted behind the robot arm looking over the shoulder from where it captured 640 × 480 RGB-D images for each inference.

**Figure 12:**
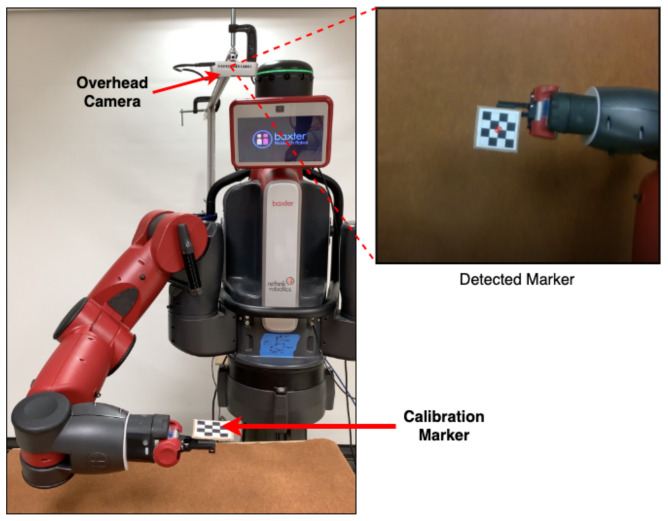
Setup for hand-eye calibration procedure.

The statically mounted overlooking camera is localized with respect to the robot frame using an automatic calibration task developed in the control module. Figure 12 shows the setup used to perform the calibration procedure. The camera detects the location of the checkerboard pattern marker mounted on the robot TCP and optimizes the extrinsics as the robot’s arm moves over a predefined grid of 3D locations in the camera’s field of view. The procedure generates transformations $T_{rc}$ and $T_{ci}$, which are used to convert the grasp poses in image frame ($G_{i}$) to robot’s frame of reference ($G_{r}$).

**Figure 13:**
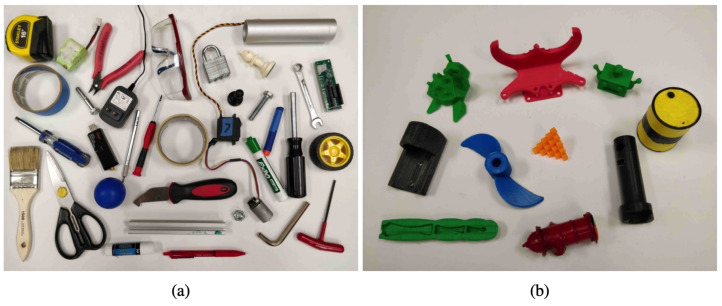
Objects used for robotic grasping experiments. (**a**) Household test objects. (**b**) Adversarial test objects.

A total of 35 household objects were chosen for testing the performance of our system. Each object was tested individually for 10 different positions and orientations which resulted in 350 grasp attempts. The objects were chosen such that each object represented a different shape, size, and geometry; and had minimum or no resemblance with each other. We created a mix of deformable, difficult to grasp, reflective, and small objects that need high precision. Figure 13a shows the set of household objects that were used for the experiments.

Another set consisting of 10 adversarial objects with complex geometry was used to evaluate the accuracy of our proposed system. These 3D printed objects have abstract geometry with indefinite surfaces and edges that are hard to perceive and grasp. Each of these objects was tested in isolation for 10 different orientations and positions and made up of a total of 100 grasp attempts. Figure 13b shows the adversarial objects used during the experiments.

Grasp poses predicted by the inference module are used to execute the grasps in an open-loop using a pick and place task. This task plans and executes open-loop collision-free trajectories considering the robot’s arm motion for planning the trajectory towards a perch position with the gripper tip aligned with and approximately 15 cm above the grasp pose $G_{r}$. The arm then moves vertically down until it reaches the required grasp pose, or a collision is detected by the robot using the force feedback. The robot then closes the antipodal gripper and moves back to the perch position. A grasp is successful if the robot lifts the object in the air at the perch position 15 cm above the grasp pose.

### 7.4. Real-World Experiments

Industrial applications such as warehouses require objects to be picked in isolation as well as from clutter. To understand how well our model trained on the Cornell dataset generalizes to novel objects, we performed grasping experiments with household and adversarial objects in isolation and clutter. For the experiment with objects in isolation, each object was tested for 10 different positions and orientations. The robot performed 334 successful grasps of the total 350 grasp attempts on household objects resulting in a grasp success rate of 95.4%, and 93 successful grasps out of 100 grasp attempts on adversarial objects giving a grasp success rate of 93%.

**Figure 14:**
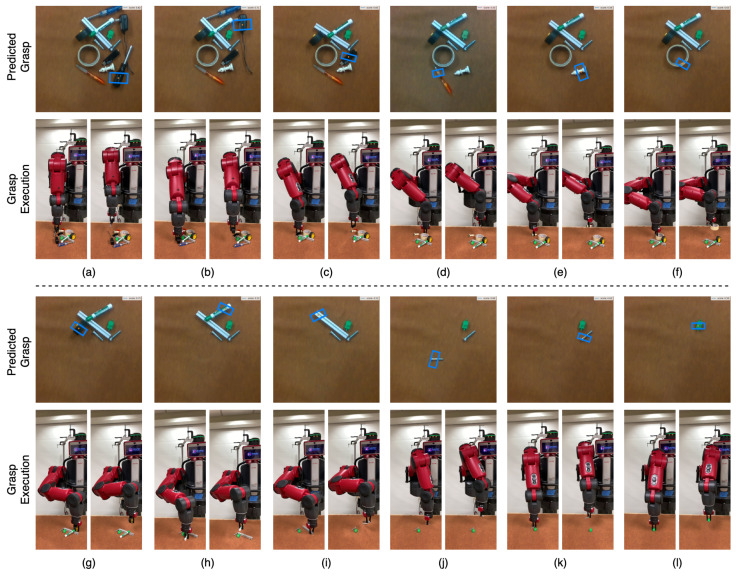
Visualization of the household objects clutter scene removal task in the real world using our GR-Convnet model trained on Cornell dataset. See attached video for a complete run. (**a**–**l**) show the grasp pose generated by inference module (top), robot grasping the object (bottom left), and robot retracting after successful grasp (bottom right) for each object.

**Figure 15:**
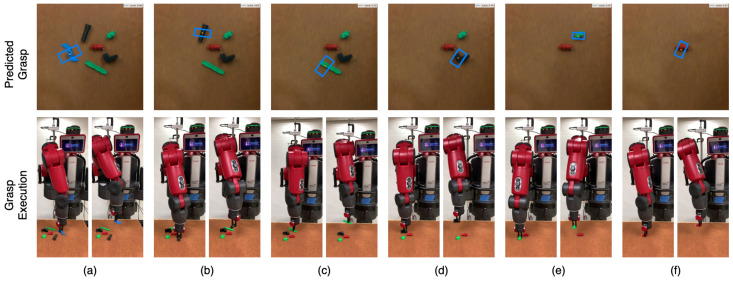
Visualization of the adversarial objects clutter scene removal task in the real world using our GR-Convnet model trained on Cornell dataset. See attached video for a complete run. (**a**–**f**) show the grasp pose generated by inference module (top), robot grasping the object (bottom left), and robot retracting after successful grasp (bottom right) for each object.

To evaluate the performance of our system for cluttered objects, we carried out multiple trials with a set of 10 to 15 distinct objects for each run. The objects were shaken in a box and emptied into a pile in front of the robot to create a cluttered scene. The robot continuously attempted to grasp and remove the object from the scene after a successful grasp. Each run was terminated when there were no objects in the camera’s field of view. An example of this is shown in Figure 14 for household objects and in Figure 15 for adversarial objects. Each run was performed without object replacement, and we recorded a mean grasp success rate of 93.5% on household object clutter and 91.0% on adversarial object clutter. This shows our method’s ability to maintain a high level of accuracy when grasping from a clutter of multiple objects. We believe that accurate grasping width prediction by GR-ConvNet for the antipodal gripper was the key reason behind the high success rate in cluttered scenes.

Despite the model being trained only on isolated objects in the Cornell dataset, we observed that it was able to efficiently predict grasps for objects in clutter. A comparison of the results for our approach compared with other deep learning-based approaches in robotic grasping is shown in Table 8. These results indicate that GR-ConvNet can effectively generalize to new objects that it has never seen before. Moreover, we can see the robustness of GR-ConvNet as it is able to predict antipodal grasps for multiple objects in a cluttered scene with high accuracy of 93.5%. The performance of GR-ConvNet in isolated scenarios is comparable to CTR [60] and DexNet 2.0 [33] for household and adversarial objects, respectively. The performance reported for our work is statically more meaningful as our sample size is 8 times more as compared to [60]. Meanwhile, we can also notice that GR-ConvNet reaches the best grasp success rate in cluttered scenarios.

### 7.5. Failure Case Analysis

In our experimental results, there are only a few cases that can be accounted for as failures. Of them, the objects that had extremely low grasp scores and those that slipped from the gripper in spite of the gripper being closed were the most common ones. This could be attributed to the inaccurate depth information coming from the camera and the gripper misalignment due to collision between the gripper and nearby objects.

Another case where the model was unable to produce a good grasp was for a transparent bottle. This could be due to inaccurate depth data captured by the camera because of possible object reflections. However, by combining depth data along with RGB data, the model was still able to generate a fairly good grasp for the transparent objects.

## 8. Multi-Step Tasks Using GR-ConvNet

To demonstrate the generalizability of the proposed GR-ConvNet to various manipulation tasks, we evaluate it on the Ravens-10 benchmark tasks presented by Zeng et al. in [10]. The Ravens-10 benchmark consists of a wide variety of vision-based multi-step manipulation tasks such as stacking a pyramid of blocks, manipulating deformable ropes, assembling kits with unseen objects, and pushing piles of small objects with closed-loop feedback. We replace the 43-layer feed-forward residual networks for picking and placing by the proposed GR-ConvNet in the Transporter-based framework [10] and train the models using behavior cloning.

### 8.1. Experimental Setup

**Figure 16:**
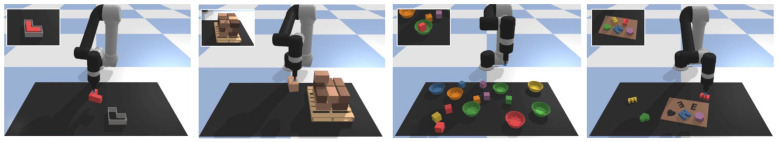
Examples of Ravens-10 benchmark tasks. Left to right: block insertion, palletizing boxes, place red in green, assembling kits.

An open-source simulation environment by Zeng et al. in [10] is used for a fair comparison with baselines. The simulated environment is built with PyBullet [59], which consists of a UR5e robot with a suction gripper overlooking the robot workspace with three RGB-D cameras pointing towards the workspace for improved visual coverage. Examples of four of the Ravens-10 benchmark tasks are shown in Figure 16. For each task, objects are randomly spawned in the robot’s workspace and the agent acts with motion primitives (pick, push or place) parameterized by a sequence of two end effector poses. The task is completed when the agent receives a reward of 1 from the reward function that comes with each task. A partial reward is given during tasks for tasks that require multiple actions to be completed.

Examples of the Ravens-10 benchmark tasks are shown in Figure 16. The goal of each task is described as follows:

(a) block-insertion: pick up the L-shaped red block and place it into the L-shaped fixture.
(b) place-red-in-green: pick up the red blocks and place them into the green bowls amidst other objects.
(c) towers-of-hanoi: sequentially move disks from one tower to another—only smaller disks can be on top of larger ones.
(d) align-box-corner: pick up the randomly sized box and align one of its corners to the L-shaped marker on the tabletop.
(e) stack-block-pyramid: sequentially stack 6 blocks into a pyramid of 3-2-1 with rainbow-colored ordering.
(f) palletizing-boxes: pick up homogeneous fixed-sized boxes and stack them in transposed layers on the pallet.
(g) assembling-kits: pick up different objects and arrange them on a board marked with corresponding silhouettes.
(h) packing-boxes: pick up randomly sized boxes and place them tightly into a container.
(i) manipulating-rope: rearrange a deformable rope such that it connects the two endpoints of a 3-sided square.
(j) sweeping-piles: push piles of small objects into a target goal zone marked on the tabletop.

### 8.2. Results

The Ravens-10 benchmark tasks are difficult as most methods tend to over-fit to the training demonstration and generalize poorly with less than 100 demonstrations. The performance is evaluated using the same metric from 0 (failure) to 100 (success) as in [10]. For each task, we report the result averaged over 100 unseen test runs trained with 1, 10, 100 and 1000 demonstrations. The performance results in Table 9 show that the GR-ConvNet-based Transporter framework can achieve state-of-the-art performance in terms of success rate on Ravens-10 benchmark tasks. While other methods require a hundred or thousand demonstrations to achieve a task success rate of over 90% for tasks such as *packing-boxes* and *sweeping-piles*, GR-ConvNet requires less than 1/10th of the number of demonstrations. This validates that the sampling efficiency of GR-ConvNet is extremely impressive when evaluated on unseen test settings. These results are consistent with our antipodal grasping experiments and demonstrate how GR-ConvNet generalizes across completely different manipulation tasks.

## 9. Discussion and Conclusions

We presented a modular solution for grasping novel objects using our improved GR-ConvNet that uses n-channel input data to generate images that can be used to infer grasp rectangles for each pixel in an image. The lightweight nature of our model makes it computationally less expensive and much faster compared to similar grasp prediction techniques. We evaluated the GR-ConvNet on two standard datasets, the Cornell grasp dataset and the Jacquard dataset, and obtained state-of-the-art results on both datasets. Additionally, to test the robustness of our network, we used stricter IoU thresholds and obtained consistently outstanding results on all Jaccard thresholds for the Cornell Dataset. Furthermore, we performed ablation studies to evaluate the effect of all individual parameters and components in our model. With the help of these experiments, we were able to identify the effect of adding dropout layers which further improved the performance of our network along with choosing the correct filter size (k) and examining the performance of our network on multiple input modalities.

We also validated the proposed system on novel real objects including household objects and adversarial objects in clutter by performing experiments using a robotic arm. The results demonstrate that our system can predict and perform accurate grasps for previously unseen objects. Moreover, the low inference time of our model makes the system suitable for closed-loop robotic grasping. Furthermore, we performed several experiments on cluttered scene removal to show that our system is capable to transfer in any industrial scenario and achieved exceptional results even though the model was trained only on singular objects.

In addition to the inference speed, using RGB-D images also simplifies the data, as it is in fewer dimensions and is easier to handle and modify. However, this increased speed also comes at a cost. The 2D representation of an object is flat compared to a point cloud. Therefore, the objects are only seen from one viewpoint, making it hard to determine the rotation of the gripper in space. Although GR-ConvNet was used to predict 4D grasps using RGB-D images in this work, it can potentially be extended to 6 DoF grasping in future work. The proposed GR-ConvNet model can also be used to explore manipulation tasks that require high precision. Another idea is to apply depth prediction techniques [63] to accurately predict depth for reflective objects, which can aid in improving the grasp prediction accuracy for reflective objects such as the bottle as discussed in Section 7.5.

## Figures and Tables

**Table 1:** A comparison of related work.

Author	Cornell Results	Jacquard Results	Graspnet Results	Real Robot	Household Objects	Adversarial Objects	Cluttered Objects	Code Available
Lenz et al. [1]	**✓**	**✗**	**✗**	**✓**	**✓**	**✗**	**✗**	**✓**
Redmon et al. [2]	**✓**	**✗**	**✗**	**✗**	**✗**	**✗**	**✗**	**✗**
Wang et al. [12]	**✓**	**✗**	**✗**	**✗**	**✗**	**✗**	**✗**	**✗**
Kumra et al. [4]	**✓**	**✗**	**✗**	**✗**	**✗**	**✗**	**✗**	**✗**
Depierre et al. [6]	**✓**	**✓**	**✗**	**✓**	**✓**	**✗**	**✗**	**✗**
Chu et al. [13]	**✓**	**✗**	**✗**	**✓**	**✓**	**✗**	**✗**	**✓**
Zhou et al. [14]	**✓**	**✓**	**✗**	**✗**	**✗**	**✗**	**✗**	**✗**
Asif et al. [15]	**✓**	**✗**	**✗**	**✓**	**✓**	**✗**	**✓**	**✗**
Morrison et al. [9]	**✓**	**✗**	**✗**	**✓**	**✓**	**✓**	**✓**	**✓**
Zhang et al. [16]	**✓**	**✓**	**✗**	**✓**	**✓**	**✗**	**✗**	**✗**
Ours	**✓**	**✓**	**✓**	**✓**	**✓**	**✓**	**✓**	**✓**

**Table 2:** Summary of antipodal robotic grasping datasets.

Dataset	Modality	Type	Objects	Images	Grasps
Cornell [5]	RGB-D	Real	240	1035	8k
Multi-Object [13]	RGB-D	Real	-	96	2.9k
Jacquard [6]	RGB-D	Sim	11k	54k	1.1M
Dexnet [33]	Depth	Sim	1500	6.7M	6.7M
VR-Grasping [43]	RGB-D	Sim	101	10k	4.8M
VMRD [16]	RGB	Real	100	4.6k	100k
Graspnet [7]	RGB-D	Real	88	97k	1.2B

**Table 3:** Comparative results on Cornell grasping dataset.

Authors	Algorithm	Accuracy (%) with IoU > 25%		Speed (ms)
		IW	OW	
Jiang et al. [5]	Fast Search	60.5	58.3	5000
Lenz et al. [1]	SAE, struct. reg.	73.9	75.6	1350
Redmon et al. [2]	AlexNet, MultiGrasp	88.0	87.1	76
Wang et al. [12]	Two-stage closed-loop	85.3	-	140
Asif et al. [52]	STEM-CaRFs	88.2	87.5	-
Kumra et al. [4]	ResNet-50x2	89.2	88.9	103
Guo et al. [32]	ZF-net	93.2	89.1	-
Zhou et al. [14]	FCGN, ResNet-101	97.7	96.6	117
Asif et al. [15]	GraspNet	90.2	90.6	24
Chu et al. [13]	Multi-grasp Res-50	96.0	96.1	120
Morrison et al. [53]	GG-CNN	73.0	69.0	**19**
Morrison et al. [9]	GG-CNN2	84.0	82.0	20
Karaoguz et al. [54]	GRPN	88.7	-	200
Zhang et al. [16]	ROI-GD, ResNet-101	93.6	93.5	39
Wang et al. [55]	DD-Net, Hourglass	97.2	96.1	-
Shi et al. [56]	EDINet-RGBD	97.7	96.6	25
Yu et al. [57]	SE-ResUNet	98.2	97.1	25
	GR-ConvNet	97.7	96.6	20
Ours	GR-ConvNet v2	**98.8**	**97.7**	20

**Table 4:** Grasp prediction accuracy (%) for Cornell dataset at different Jaccard thresholds.

Approach	IoU > 25%	IoU > 30%	IoU > 35%	IoU > 40%
Guo et al. [32]	93.2	91.0	85.3	-
Chu et al. [13]	96.0	92.7	87.6	82.6
Wang et al. [58]	94.4	92.8	90.2	85.7
Shi et al. [56]	97.7	97.6	97.1	96.5
Ours	**98.8**	**98.8**	**98.8**	**96.6**

**Table 5:** Comparative results on the Jacquard dataset.

Authors	Algorithm	Accuracy (%)	
		IoU	SGT
Depierre et al. [6]	AlexNet	74.2	72.4
Morrison et al. [9]	GG-CNN2	84.0	85.0
Zhou et al. [14]	FCGN, ResNet-101	92.8	81.9
Zhang et al. [16]	ROI-GD, ResNet-101	93.6	-
Wang [55]	DD-Net, Hourglass	**97.0**	89.4
Yu et al. [57]	SE-ResUNet	95.7	-
	GR-ConvNet (b = 8, d = 0.0)	94.6	89.5
Ours	GR-ConvNet v2 (b = 16, d = 0.1)	95.1	**91.4**

**Table 6:** Comparative results for grasp prediction accuracy (%) on Graspnet 1-billion dataset for different validation splits.

Approach	5-Fold	Seen	Similar	Novel
GGCNN [9]	82.3	83.0	79.4	76.3
Multi-grasp [13]	86.0	82.7	77.8	72.7
GR-ConvNet (k = 32, d = 0.0)	96.1	96.2	94.8	87.9
GR-ConvNet v2 (k = 32, d = 0.1)	**98.7**	**97.9**	**96.0**	**90.5**

**Table 7:** Pick success rate (%) on YCB objects in simulation.

Approach	Training Dataset	Isolated	Cluttered
GGCNN	Cornell	79.0 *	74.5 *
GGCNN	Jacquard	85.5 *	82.0 *
GR-ConvNet v2	Cornell	**98.0**	92.0
GR-ConvNet v2	Jacquard	97.5	**96.5**

* The accuracy is calculated using the open source code and model.

**Table 8:** Comparative results for grasp success rate (%) in real-world for different scenarios.

Approach	Training Dataset	Household Isolated	Adversarial Isolated	Household Cluttered	Adversarial Cluttered
SAE, struct. reg. [1]	Cornell	89.0 (89/100)	-	-	-
Alexnet based CNN [3]	Custom	66.0 (99/150)	-	38.4 (50/130)	-
Robust Best Grasp [36]	ModelNet in Sim	80.0 (80/100)	-	-	-
Multi-grasp Res-50 [13]	Cornell	89.0 (89/100)	-	-	-
DexNet 2.0 [33]	DexNet in Sim	80.0 (40/50)	92.5 (74/80)	-	-
GPD [61]	CAD models	-	-	77.3 (116/138)	-
CTR [60]	Custom in OpenRAVE	**97.5** (39/40)	-	88.8 (66/74)	-
GGCNN [9]	Cornell	91.6 (110/120)	83.7 (67/80)	86.4 (83/96)	-
GR-ConvNet	Cornell	95.4 (334/350)	**93.0** (93/100)	**93.5** (187/200)	**91.0** (91/100)

**Table 9:** GR-ConvNet performance on Ravens-10 benchmark tasks. Task success rate (mean %) vs. demonstration used in training.

	**align-box-corner**				**assembling-kits**				**block-insertion**				**manipulating-rope**				**packing-boxes**			
Approach	1	10	100	1000	1	10	100	1000	1	10	100	1000	1	10	100	1000	1	10	100	1000
GR-ConvNet	**62.0**	**91.0**	**100**	**100**	**56.8**	**83.2**	**99.4**	**99.6**	**100**	**100**	**100**	**100**	**25.7**	**87.0**	**99.9**	**100**	**96.1**	**99.9**	**99.9**	**100**
Transporter [10]	35.0	85.0	97.0	98.3	28.4	78.6	90.4	94.6	**100**	**100**	**100**	**100**	21.9	73.2	85.4	92.1	56.8	58.3	72.1	81.3
Form2Fit [62]	7.0	2.0	5.0	16.0	3.4	7.6	24.2	37.6	17.0	19.0	23.0	29.0	11.9	38.8	36.7	47.7	29.9	52.5	62.3	66.8
	**palletizing-boxes**				**place-red-in-green**				**stack-block-pyramid**				**sweeping-piles**				**towers-of-hanoi**			
	1	10	100	1000	1	10	100	1000	1	10	100	1000	1	10	100	1000	1	10	100	1000
GR-ConvNet	**84.2**	**98.2**	**100**	**100**	**92.3**	**100**	**100**	**100**	**23.5**	**79.3**	**94.6**	**97.1**	**98.2**	**99.1**	**99.2**	**98.9**	**98.2**	**99.9**	**99.9**	**99.9**
Transporter [10]	63.2	77.4	91.7	97.9	84.5	**100**	**100**	**100**	13.3	42.6	56.2	78.2	52.4	74.4	71.5	96.1	73.1	83.9	97.3	98.1
Form2Fit [62]	21.6	42.0	52.1	65.3	83.4	**100**	**100**	**100**	19.7	17.5	18.5	32.5	13.2	15.6	26.7	38.4	3.6	4.4	3.7	7.0

## Data Availability

We open-sourced the implementation of the proposed inference module at https://github.com/skumra/robotic-grasping (accessed on 15 August 2022) and control module at https://github.com/skumra/baxter-pnp (accessed on 15 August 2022).
